# Associations Between Childbirth-Related Posttraumatic Stress Symptoms and Mother–Infant Bonding Difficulties: The Role of Maternal Stress and Perceived Infant Regulatory Difficulties

**DOI:** 10.3390/jcm15197415

**Published:** 2026-09-24

**Authors:** Ana Beato, Sara Albuquerque, Cláudia P. Pires, Stephanie Alves

**Affiliations:** 1 HEI-Lab: Digital Human-Environment Interaction Labs, Lusófona University, 1749-024 Lisbon, Portugal; sara.albuquerque@ulusofona.pt (S.A.); claudia.pires@ulusofona.pt (C.P.P.); stephanie.alves@ulusofona.pt (S.A.); 2 Instituto Superior Miguel Torga, 3000-132 Coimbra, Portugal; 3 Center for Research in Neuropsychology and Cognitive and Behavioral Intervention (CINEICC), University of Coimbra, 3000-115 Coimbra, Portugal

**Keywords:** childbirth-related posttraumatic stress symptoms, mother–infant bonding, maternal stress, perceived infant regulatory difficulties, postpartum, maternal mental health

## Abstract

**Background**: Childbirth-related posttraumatic stress symptoms (CB-PTSS) have been associated with impaired mother–infant bonding, but the factors underlying this relationship remain unclear. This study examined whether maternal stress and perceived infant regulatory difficulties (PIRD) independently accounted for the association between CB-PTSS and mother–infant bonding difficulties in a statistical mediation model. **Methods**: This cross-sectional study included 542 Portuguese mothers of infants aged up to 24 months. Participants completed validated measures of CB-PTSS, maternal stress, perceived infant regulatory difficulties, and mother–infant bonding. Hierarchical regression and parallel mediation analyses were conducted. **Results**: Higher CB-PTSS were associated with greater maternal stress, more perceived infant regulatory difficulties, and greater bonding difficulties. Perceived infant regulatory difficulties explained additional variance beyond CB-PTSS and maternal stress. Both maternal stress and infant regulatory difficulties showed significant independent indirect effects, while the direct association between CB-PTSS and bonding difficulties remained significant, indicating partial mediation. Given the cross-sectional design, these findings should be interpreted as statistical associations rather than evidence of temporal or causal relationships. Longitudinal studies are needed to establish temporal ordering and directionality. **Conclusions**: Maternal stress and perceived infant regulatory difficulties may represent key statistical correlates linking traumatic childbirth to bonding difficulties and constitute promising targets for early screening and intervention.

## 1. Introduction

The emotional connection that forms between mother and infant is central to maternal and child well-being [1,2] and is conceptually distinct from infant attachment. Attachment theory describes the infant’s biologically rooted tie to the caregiver, whereas mother–infant bonding refers to the mother’s feelings and emotional investment toward her infant, which begins during pregnancy and develops across the postpartum period [3,4,5]. Bonding difficulties are associated with maternal depression, anxiety, and stress during the first postpartum year [6] and may have longer-term implications, as early bonding difficulties have been linked to later child social-emotional problems [7]. Conversely, a healthy early relationship, supported by sensitive parenting, contributes to infant socioemotional and cognitive development [8,9]. Mother–infant bonding may therefore represent an important relational pathway through which maternal distress affects early development. Childbirth-related posttraumatic stress symptoms (CB-PTSS) constitute one potential source of such distress. A recent meta-analysis of 154 studies estimated prevalence rates of 4.7% for full childbirth-related PTSD and 12.3% for clinically significant CB-PTSS [10,11]. CB-PTSS refers to posttraumatic stress symptoms associated with the childbirth experience and is distinct from a clinical PTSD diagnosis, which requires specified symptom clusters, duration, and functional impairment. It is also conceptually distinct from postpartum depression, although the two may co-occur. Within the present study, CB-PTSS is considered a continuous indicator of symptom severity rather than a proxy for clinical diagnosis. A diathesis-stress framework suggests that pre-birth vulnerabilities, such as previous psychological difficulties and fear of childbirth, interact with the birth experience to shape traumatic appraisals, while postpartum stress and coping may contribute to symptom maintenance [12]. Similarly, Beck’s middle-range theory of traumatic childbirth proposes that birth trauma may have a “ripple effect” extending beyond maternal mental health to affect the emotional relationship between mother and infant [13]. Consistent with this perspective, postnatal PTSD symptoms have been associated with weaker parent–infant bonding independently of depressive symptoms [14]. A meta-analysis of 22 studies including 9472 participants found a moderate positive association between CB-PTSS and impaired bonding [15], while a prospective cohort of more than 3000 first-time mothers found that CB-PTSS at one-month postpartum predicted poorer bonding at one, six, and twelve months [16]. However, the mechanisms underlying this association remain unclear. The association between CB-PTSS and impaired bonding may be partly accounted for by depressive symptoms and broader psychological distress [15], and prospective findings are not consistent across studies. For example, the association between birth-related PTSD symptoms and subsequent bonding was attenuated after accounting for concurrent psychological distress in one population-based cohort [17]. Moreover, studies distinguishing general posttraumatic symptoms from birth-specific re-experiencing and avoidance have reported stronger associations for general symptoms, whereas birth-related symptoms have shown weaker or inconsistent associations with bonding [18,19]. A systematic review similarly highlighted substantial heterogeneity in samples, timing, and assessment, leaving the pattern of associations unresolved [20]. Thus, an important question is which psychological factors may account for the association between CB-PTSS and mother–infant bonding. Maternal stress represents one plausible factor. CB-PTSS may contribute to broader psychological distress and depleted coping resources [12], potentially reducing the emotional resources available for sensitive caregiving. Parenting stress is negatively associated with maternal sensitivity [21], and maternal sensitivity and responsiveness may partly account for associations between postpartum psychological symptoms and impaired bonding [22]. Persistently elevated CB-PTSS during the first four postpartum months has also been associated with reduced maternal sensitivity, which in turn was related to infant avoidance of the mother’s gaze [23]. Because sensitivity was not directly assessed in the present study, general perceived stress is considered a proximal, self-reported indicator of the emotional-resource difficulties identified in this literature. A second potentially relevant factor is perceived infant regulatory difficulty (PIRD), referring to maternal perceptions of infant sleep, crying, irritability, and soothability. Mothers who perceive their infants as more difficult to regulate tend to report poorer bonding across independent samples [24,25,26], consistent with meta-analytic evidence [27]. Importantly, maternal perceptions may not simply reflect infants’ observed regulatory behaviour. In a Norwegian pregnancy cohort, maternal representations of infant temperament, rather than observed regulatory difficulties, predicted subsequent parenting stress and depressive symptoms [28]. CB-PTSS may therefore be associated with bonding through two potentially distinct pathways: greater maternal stress and reduced emotional resources, and greater perceived difficulty in regulating the infant. The transactional model proposed by Sameroff provides a framework for understanding these pathways. It conceptualizes development as a continuous exchange among caregiver characteristics, infant characteristics, and the relational context, rather than as a unidirectional process [29,30]. Maternal characteristics influence caregiving, infant characteristics influence maternal perceptions, and these reciprocal exchanges contribute to changes in both partners and their relationship over time [31]. Applied to childbirth-related trauma, this framework allows maternal stress and PIRD to be considered as complementary components of the same developing relational system. Building on this framework, the present study investigated whether maternal stress and perceived infant regulatory difficulties independently account for the association between CB-PTSS and mother–infant bonding difficulties in a statistical mediation model. We hypothesized that higher CB-PTSS would be associated with greater mother–infant bonding difficulties, both directly and indirectly through increased maternal stress and perceived infant regulatory difficulties. We further expected these indirect associations to remain significant after adjusting for maternal age, infant age, educational level, household income, and previous psychological problems. Given the cross-sectional design, all associations should be interpreted as statistical rather than causal or temporally ordered. CB-PTSS is treated throughout as a continuous, dimensional measure of symptom severity rather than as a categorical proxy for clinical diagnosis.

## 2. Materials and Methods

### 2.1. Participants

Sociodemographic and obstetric characteristics of the sample are presented in Table 1. The final sample comprised 542 Portuguese mothers of infants aged from 2 weeks to 24 months (*M* = 8.56 months; *SD* = 6.47, *Mdn* = 7.0). Mothers had a mean age of 32.76 years (*SD* = 5.03, *Mdn* = 33.00). Most had completed a university degree (62.7%). Household monthly income was equal to or higher than €1583 for nearly half of the sample (49.1%), and most had no prior psychological problems (*M* = 1.95, *SD* = 0.70). The large majority were married, in a civil union, or in a relationship (94.3%).

Regarding obstetric and reproductive history, 24.0% of mothers reported having experienced difficulty conceiving, and most participants (63.8%) were primiparous. Mean gestational age at birth was 38.89 weeks (*SD* = 1.54, *Mdn* = 39). Just over half of participants (52.2%) reported a vaginal delivery. Obstetric complications during pregnancy or childbirth were reported by 56.6% of participants.

### 2.2. Measures

#### 2.2.1. Sociodemographic and Clinical Questionnaire

Participants completed a study-specific questionnaire designed to collect sociodemographic, obstetric, and clinical information. Sociodemographic variables included maternal age, educational level, household income, relationship status, employment status, parity, previous psychological problems, and infant age. Obstetric and postpartum information comprised difficulty in conceiving, infant age, gestational age, type of delivery, and obstetric complications.

#### 2.2.2. Childbirth-Related Posttraumatic Stress Symptoms (CB-PTSS)

Modified Perinatal Posttraumatic Stress Disorder Questionnaire. CB-PTSS were assessed using the Modified Perinatal Posttraumatic Stress Disorder Questionnaire (PPQ-II; [32]). It comprises 14 items assessing posttraumatic stress symptoms following childbirth across three symptom domains: intrusion (e.g., nightmares about childbirth), avoidance and emotional numbing (e.g., forgetting aspects of the hospitalization), and hyperarousal (e.g., irritability or anger). Items are rated on a 5-point Likert scale ranging from 0 (*not at all*) to 4 (*frequently for more than one month*).

The present study used the European Portuguese version of the PPQ-II, which comprises 11 items, following its psychometric validation in Portuguese postpartum women [33]. A total score was obtained by summing all the items (possible range: 0–44), with higher scores reflecting greater severity of CB-PTSS. In the present sample, the instrument demonstrated good internal consistency (Cronbach’s α = 0.85).

#### 2.2.3. Mother–Infant Bonding

Postpartum Bonding Questionnaire. The quality of mother–infant bonding was assessed using the Portuguese short version of the Postpartum Bonding Questionnaire (PBQ) [34,35,36]. The PBQ is a 12-item self-report measure designed to assess mothers’ feelings and attitudes toward their infant (e.g., “I feel distant from my baby” and “I love to cuddle my baby”). Participants rated the extent to which each statement reflected their experience using a 6-point Likert scale ranging from 0 (always) to 5 (never), with positively worded items reverse-scored. A total score is obtained by averaging the items (possible range: 0–5). Higher scores indicate greater difficulties in mother–infant bonding.

In the Portuguese validation study, six confirmatory factor analysis models, based on previous PBQ research, were tested [36]. The model that demonstrated the best fit consisted of a 12-item unidimensional structure corresponding to the first factor of the original instrument, labeled *impaired mother–infant bonding* [34,35]. This version showed good internal consistency and temporal stability, as well as adequate convergent and discriminant validity. In the present study, the PBQ demonstrated acceptable internal consistency (Cronbach’s α = 0.75).

#### 2.2.4. Maternal Stress

Depression Anxiety Stress Scales. Stress symptoms were assessed using the Stress subscale of the Portuguese version of the Depression Anxiety Stress Scales-21 (DASS-21) [37,38]. The DASS-21 is a 21-item self-report instrument comprising three 7-item subscales assessing symptoms of depression, anxiety, and stress. In the present study, only the stress subscale was used. This subscale evaluates persistent tension and negative affective states, including difficulty relaxing, nervous arousal, irritability, agitation, and impatience (e.g., “I found it difficult to relax”). Depression and anxiety symptoms were not assessed as separate outcome variables in the present study. Participants rated the extent to which each statement applied to them over the previous week using a 4-point Likert scale ranging from 0 (*did not apply to me at all*) to 3 (*applied to me very much, or most of the time*). Stress scores were calculated by summing the seven items (possible range: 0–21). Higher scores indicate greater levels of perceived stress. The Portuguese version of the DASS-21 retains the original three-factor structure and has demonstrated good psychometric properties [38]. In the present study, the stress subscale showed excellent internal consistency (Cronbach’s α = 0.90).

#### 2.2.5. Perceived Infant Regulatory Difficulties (PIRD)

Perceived infant regulatory difficulties (PIRD) were assessed using four maternal self-report items evaluating common infant regulatory challenges during the postpartum period, and developed based on previous literature [39,40]: (1) infant sleep problems (“I am experiencing problems with my baby’s sleep”), (2) infant irritability/agitation (“My baby is irritable and/or agitated”), (3) frequent crying (“My baby cries a lot”), and (4) difficulty being soothed (“My baby is difficult to soothe or calm”). Responses were rated on a 5-point Likert scale ranging from 1 (*strongly disagree*) to 5 (*strongly agree*), with response options of *strongly disagree*, *disagree*, *neither agree nor disagree*, *agree*, and *strongly agree*. A total score was obtained by summing the four items (possible range: 4–20), with higher scores indicating greater perceived infant regulatory difficulties. The four items were moderately to strongly intercorrelated (Pearson’s *r* = 0.37–0.73, all *p*s < 0.001), and the resulting composite demonstrated good internal consistency (Cronbach’s α = 0.81).

### 2.3. Procedure

The present study was conducted as part of a broader research project examining psychosocial risk and protective factors associated with maternal adjustment during the transition to motherhood. The study protocol was reviewed and approved by the Ethics and Deontology Committee of the School of Psychology and Life Sciences, Universidade Lusófona. All participants provided electronic informed consent before participation.

Data were collected between February and March 2020 using an online survey hosted on Typeform. Participants were recruited through announcements posted in social media groups for mothers. The announcements provided information about the study and invited eligible mothers to participate. Interested participants accessed the survey through the provided link and completed the study measures independently and remotely. A dedicated email address for the research project was also provided, allowing potential and enrolled participants to request additional information and clarify any questions regarding the study or participation procedures.

Eligibility criteria were as follows: (a) being the biological mother of a singleton infant younger than 24 months; (b) having conceived the child within a heterosexual relationship; (c) being at least 18 years of age; and (d) having sufficient proficiency in Portuguese to complete the study questionnaires. Participants who met the eligibility criteria completed the online assessment following electronic informed consent. As all study procedures were conducted remotely, there were no face-to-face study visits, and all assessment measures were self-administered by participants; no researcher-administered clinical assessments were conducted. Participation was voluntary, and no financial compensation was provided.

A total of 560 mothers participated in the study. Eighteen participants were excluded because more than 20% of the items were missing on one or more study measures. The final analytical sample therefore comprised 542 participants.

### 2.4. Statistical Analyses

Statistical analyses were performed using IBM SPSS Statistics for Windows, Version 31.0 (IBM Corp., Armonk, NY, USA). Mediation analyses were conducted using the PROCESS macro for SPSS, version 5.0 [41]. A sensitivity power analysis was conducted using G*Power 3.1, based on the final sample (*N* = 542), α = 0.05, and three predictors included in the regression models, indicating adequate power (>0.95) to detect small effect sizes.

Descriptive statistics were computed to characterize the sample and summarize the distribution of the study variables. Normality was assessed through skewness and kurtosis values, with coefficients between −2 and 2 considered indicative of an acceptable distribution [42]. Although some variables showed moderate positive kurtosis, given the large sample size (*N* = 542) and the reliance on bootstrap-based resampling procedures for the mediation analyses, which do not assume normality of the sampling distribution of indirect effects, these deviations were not considered to compromise the validity of the results. Missing data were handled through listwise deletion, and statistical significance was set at *p* < 0.05 (two-tailed).

Pearson’s correlation coefficients were computed to examine bivariate associations among the study variables (CB-PTSS, maternal stress, perceived infant regulatory difficulties, and mother–infant bonding difficulties) and the covariates included in the statistical mediation model (maternal age, educational level, household income, infant age, and previous psychological problems). Correlation magnitudes were interpreted as small (*r* ≈ 0.10), medium (*r* ≈ 0.30), or large (*r* ≈ 0.50) [43].

A hierarchical multiple linear regression analysis was conducted to examine the unique statistical contribution of CB-PTSS, maternal stress, and perceived infant regulatory difficulties to mother–infant bonding difficulties, while controlling for relevant covariates. Maternal age, educational level, household income, infant age, and previous psychological problems were entered in the first block. CB-PTSS and maternal stress were entered in the second block, followed by perceived infant regulatory difficulties in the third block. Standardized regression coefficients (β) and changes in explained variance (ΔR^2^) were examined for each step. Model assumptions were verified through inspection of residual independence (Durbin–Watson statistic) and multicollinearity diagnostics, with tolerance values above 0.20 and variance inflation factors (VIFs) below 5 considered acceptable [44].

The hypothesized statistical mediation model was tested using the PROCESS macro for SPSS (Version 5.0; Model 4) [41], controlling for maternal age, educational level, household income, infant age, and previous psychological problems, selected a priori based on previous evidence associating these variables with maternal psychological adjustment and mother–infant bonding. CB-PTSS were specified as the independent variable, mother–infant bonding difficulties as the dependent variable, and maternal stress and perceived infant regulatory difficulties as parallel statistical mediators. Indirect effects were estimated using 5000 bias-corrected bootstrap resamples, and statistical significance was determined by 95% bias-corrected bootstrap confidence intervals (95% CIs). Indirect effects were considered statistically significant when the corresponding confidence interval did not include zero. As this study employed a cross-sectional design, the terms “mediator,” “indirect effect,” and “direct effect” are used here in their statistical sense to describe the pattern of associations tested, and do not imply a confirmed causal or temporal sequence among the study variables.

## 3. Results

### 3.1. Descriptive Statistics and Correlations

Pearson’s correlation coefficients among the study variables and covariates are presented in Table 2. CB-PTSS, maternal stress, perceived infant regulatory difficulties, and mother–infant bonding difficulties were all positively and significantly associated. The strongest association was observed between CB-PTSS and maternal stress, followed by the association between perceived infant regulatory difficulties and mother–infant bonding difficulties. Previous psychological problems before or during pregnancy were positively associated with all primary study variables, whereas maternal age showed no significant associations with any of them. Infant age was weakly associated with maternal stress, perceived infant regulatory difficulties, and mother–infant bonding difficulties, but not with CB-PTSS. Educational level and household income were positively associated with mother–infant bonding difficulties, while both educational level and household income were negatively associated with previous psychological problems.

### 3.2. Hierarchical Regression Analysis

A hierarchical multiple regression was conducted to examine the unique statistical contribution of CB-PTSS, maternal stress, and perceived infant regulatory difficulties on mother–infant bonding difficulties while controlling for maternal age, educational level, household income, infant age, and previous psychological problems (Table 3). In Block 1, educational level, household income, infant age, and previous psychological problems were significant predictors of mother–infant bonding difficulties, whereas maternal age was not. After entering CB-PTSS and maternal stress in Block 2, both variables emerged as significant positive predictors, while the effect of previous psychological problems was no longer statistically significant. In the final model, perceived infant regulatory difficulties was the strongest predictor of bonding difficulties (*β* = 0.44, *p* < 0.001), followed by maternal stress (*β* = 0.18, *p* < 0.001) and CB-PTSS (*β* = 0.15, *p* = 0.001), all three remaining statistically significant. Educational level (*β* = 0.10, *p* = 0.009) and household income (*β* = 0.13, *p* < 0.001) also remained significant predictors of bonding difficulties in the final model, whereas maternal age, infant age, and previous psychological problems were no longer significant once CB-PTSS, maternal stress, and perceived infant regulatory difficulties were entered.

### 3.3. Mediation Analysis

CB-PTSS significantly predicted both maternal stress (a1 = 0.29, *p* < 0.001) and perceived infant regulatory difficulties (a2 = 0.02, *p* < 0.001). In turn, both maternal stress (b1 = 0.18, *p* < 0.001) and perceived infant regulatory difficulties (b2 = 1.96, *p* < 0.001) significantly predicted mother–infant bonding difficulties (see Figure 1 and Table 4). These models explained 47.6% of the variance in maternal stress, *R*^2^ = 0.48, *F*(6, 527) = 79.84, *p* < 0.001, and 8% of the variance in perceived infant regulatory difficulties, *R*^2^ = 0.08, *F*(6, 527) = 7.61, *p* < 0.001. The full model explained 41% of the variance in mother–infant bonding difficulties, *R*^2^ = 0.41, *F*(8, 525) = 45.52, *p* < 0.001. Among the covariates, infant age and previous psychological problems were significantly associated with both maternal stress and perceived infant regulatory difficulties. In contrast, educational level and household income were significantly associated with mother–infant bonding difficulties.

The total effect of CB-PTSS on mother–infant bonding difficulties was significant (c = 0.17, *p* < 0.001, 95% CI [0.125, 0.212]), as was the direct effect after accounting for both mediators (c′ = 0.08, *p* = 0.001, 95% CI [0.032, 0.124]), indicating partial mediation. The total indirect effect was significant (*B* = 0.090, 95% CI [0.050, 0.131]). Examination of the specific indirect effects revealed that both maternal stress (*B* = 0.051, 95% CI [0.020, 0.084]) and perceived infant regulatory difficulties (*B* = 0.039, 95% CI [0.016, 0.064]) independently and significantly mediated the association between CB-PTSS and mother–infant bonding difficulties. The full model coefficients, including all covariates, are presented in Table 4.

## 4. Discussion

The present study examined whether maternal stress and perceived infant regulatory difficulties statistically accounted for the association between CB-PTSS and mother–infant bonding difficulties. CB-PTSS, as assessed here, is conceptually distinct from postpartum depression and postpartum anxiety, neither of which was measured as a separate outcome in the present study; references below to “psychological distress” or “depressive symptoms” describe findings from the cited literature, not variables assessed in this sample. As expected, mothers reporting higher levels of CB-PTSS also reported more bonding difficulties with their infant. This association co-occurred with higher maternal stress and by greater perceived infant regulatory difficulties, although the direct association between CB-PTSS and bonding remained significant. These findings suggest that childbirth-related traumatic stress may be associated with the early mother–infant relationship through multiple co-occurring psychological factors rather than a single one. Given the cross-sectional design of the present study, these associations should not be interpreted as evidence of causal or temporally ordered relationships.

The association between CB-PTSS and impaired bonding is consistent with previous studies and recent meta-analyses showing that mothers experiencing traumatic stress after childbirth tend to report greater emotional distance from their infants and more difficulties establishing a positive emotional relationship [15,45]. Our findings extend this literature by showing that this association is not accounted for solely by traumatic symptoms themselves. Instead, maternal stress and the way mothers perceive their infants’ regulatory behaviours appear to account for part of this relationship. This supports the idea that the consequences of childbirth-related trauma extend beyond the traumatic experience itself and are associated with the mother’s day-to-day interactions with her infant.

Maternal stress emerged as one of the factors statistically associated with the link between CB-PTSS and bonding difficulties. This finding is in line with previous evidence showing that women experiencing post-traumatic stress symptoms often report greater psychological distress and reduced emotional availability during the postpartum period [12]. Persistent stress may be associated with mothers’ capacity to respond sensitively to infant cues, making positive interactions more difficult and relating to the development of the emotional bond. Previous research has consistently shown that higher parenting stress is associated with lower maternal sensitivity [21] and that maternal responsiveness is associated, in part, with the association between postpartum psychological difficulties and impaired bonding [22]. Our findings are consistent with this body of work and suggest that maternal stress is one factor statistically associated with childbirth-related trauma and the early mother–infant relationship.

The second factor, perceived infant regulatory difficulties, represents the most novel finding of the present study. Mothers with higher CB-PTSS were more likely to perceive their infants as difficult to soothe, more irritable, more prone to crying, or experiencing sleep problems, and these perceptions were associated with greater bonding difficulties. Importantly, this effect remained significant even after accounting for maternal stress, suggesting that it reflects a statistically distinct association.

This finding should be interpreted in light of the transactional model of development [29], which proposes that the parent–infant relationship develops through continuous reciprocal interactions between parent and infant. From this perspective, maternal psychological functioning is theorized to shape how infant behaviour is interpreted, while infant behaviour is theorized to influence maternal emotional responses. It is therefore possible that mothers experiencing CB-PTSS become more sensitive to infant distress or interpret normal regulatory challenges as more difficult to manage. In turn, these perceptions may be associated with less positive interactions and contribute to bonding difficulties. Recent longitudinal evidence supports this interpretation by showing that parental perceptions of infant temperament predict subsequent parenting stress and emotional adjustment more strongly than objectively observed infant behaviour [28].

Another noteworthy finding was that perceived infant regulatory difficulties explained a substantial proportion of variance in bonding difficulties beyond CB-PTSS and maternal stress and showed the strongest statistical association with bonding difficulties in the regression model. This does not necessarily mean that infant behaviour is more important than maternal psychological functioning. Rather, it highlights the importance of maternal appraisal of infant behaviour in the early relationship. Mothers do not respond to infant behaviour alone; they respond to how they understand and interpret that behaviour. This distinction has received relatively little attention in the childbirth trauma literature and deserves further investigation.

The persistence of a significant direct effect after including both mediators indicates that other factors may also be associated with CB-PTSS and bonding. Factors such as depressive symptoms, maternal self-efficacy, reflective functioning, trauma-related avoidance, or partner support may also play an important role and should be considered in future studies. Understanding these additional factors may help clarify why some mothers experience significant bonding difficulties after traumatic childbirth whereas others remain emotionally connected to their infants despite experiencing post-traumatic stress symptoms.

An additional finding was that higher educational level and household income remained significantly associated with greater mother–infant bonding difficulties, even after controlling for CB-PTSS, maternal stress, perceived infant regulatory difficulties, and the remaining covariates. This finding was unexpected. Although socioeconomic status is often assumed to be protective for postpartum adjustment, systematic evidence for this assumption is weak: a 2019 systematic review of socioeconomic and demographic correlates of bonding found that education, employment, and income were significantly associated with bonding in only a minority of the studies that examined them, with roughly half of the significant associations favoring higher socioeconomic status and half running in the opposite direction, leading the authors to conclude that no sociodemographic correlate was consistent enough to be considered an established predictor of bonding [46]. Although counterintuitive, a positive association between higher education and greater bonding difficulties has been reported in other postpartum samples [47,48], suggesting it may not be an isolated or artifactual finding. This association should not be interpreted as evidence that higher socioeconomic status causes greater bonding difficulties; rather, it represents an association observed in this specific sample and statistical model, which may reflect residual or unmeasured confounding. Several unmeasured factors could plausibly account for this association. One possible explanation is a mismatch between role demands and available informal support: this association may reflect greater reliance on formal childcare, reduced contact with extended family and friends, or higher rates of full-time employment among more highly educated and higher-income mothers, independent of material advantage [49]. An alternative explanation is that education and income, as distinct indicators of socioeconomic status, may capture different dimensions of social position, resources, occupational circumstances, and family organization, each of which could relate differently to bonding. Other plausible unmeasured factors include the duration and characteristics of maternity or parental leave, the timing of return to employment, occupational workload and flexibility, work–family conflict, availability of childcare, access to informal social support, and differing expectations regarding motherhood. None of these factors were assessed in the present study and should be examined directly in future research. Given these limitations, this finding should be interpreted with caution and treated as an empirical observation requiring replication and mechanistic clarification, rather than as evidence of a robust or well-understood socioeconomic effect on bonding.

### 4.1. Strengths, Limitations, and Future Research

This study has several strengths that should be acknowledged. It included a large sample of postpartum mothers, providing adequate statistical power to examine the proposed parallel mediation analyses. In addition, the statistical mediation model remained significant after adjustment for relevant maternal and infant sociodemographic and clinical characteristics, strengthening the robustness of the findings. Finally, by simultaneously examining maternal stress and perceived infant regulatory difficulties as complementary correlates linking CB-PTSS to mother–infant bonding difficulties, this study offers an innovative contribution to the literature and advances understanding of the patterns of association underlying early postpartum adjustment.

Despite these strengths, several limitations should be considered. The cross-sectional design precludes conclusions regarding causal relationships and temporal direction among the study variables; the statistical mediation tested in this study does not establish that maternal stress or perceived infant regulatory difficulties causally produce, or temporally precede, bonding difficulties. Longitudinal studies with repeated assessments, ideally from pregnancy through the postpartum period, are needed to establish the temporal ordering and directionality of these associations. Also, because CB-PTSS were assessed via self-report rather than clinical interview, our findings speak to symptom severity rather than diagnosed PTSD; future work using structured diagnostic assessment would help clarify whether the associations observed here extend to clinical PTSD. Furthermore, all variables were assessed using maternal self-report measures, raising the possibility of shared method variance and response bias. In particular, infant regulatory difficulties reflected mothers’ perceptions rather than objective or observational assessments of infant behavior, which may have been influenced by maternal psychological functioning. Future research should therefore combine maternal reports with observer-rated mother–infant interaction measures, standardized observational assessments of infant regulation and temperament, and prospective infant diaries documenting crying, sleep, feeding, and other regulatory behaviors. These multi-method approaches would help distinguish objectively observed infant characteristics from maternal perceptions potentially shaped by psychological distress. In addition, depressive and anxiety symptoms were not assessed as separate constructs. Therefore, the present findings cannot establish whether the associations involving CB-PTSS are independent of overlapping postpartum depressive or anxiety symptoms. Future studies should assess these domains concurrently to clarify their unique and shared contributions to mother–infant bonding.

### 4.2. Clinical Implications

The present findings have several important clinical implications. First, they highlight the importance of routinely screening for CB-PTSS during the postpartum period, as these symptoms have been associated with not only maternal psychological well-being but also the quality of the early mother–infant relationship. Early identification of mothers experiencing CB-PTSS could facilitate timely referral to appropriate psychological support before bonding difficulties become clinically significant.

Second, the finding that maternal stress and perceived infant regulatory difficulties independently accounted for part of the association between CB-PTSS and mother–infant bonding difficulties suggests that interventions should extend beyond the treatment of trauma symptoms alone. Comprehensive postpartum interventions may benefit from integrating trauma-focused approaches with strategies aimed at reducing maternal stress and increasing parents’ understanding of infant behaviour and regulatory capacities. Supporting mothers in interpreting and responding sensitively to infant cues may reduce perceptions of infant regulatory difficulties and foster more positive mother–infant interactions.

Finally, because the association between CB-PTSS and bonding difficulties remained significant after accounting for both statistical correlates, interventions should also address the broader relational consequences statistically associated with traumatic childbirth. Relationship-based approaches that strengthen maternal sensitivity and emotional availability may complement trauma-focused treatment, supporting a more comprehensive approach to promoting healthy mother–infant bonding.

## Figures and Tables

**Figure 1:**
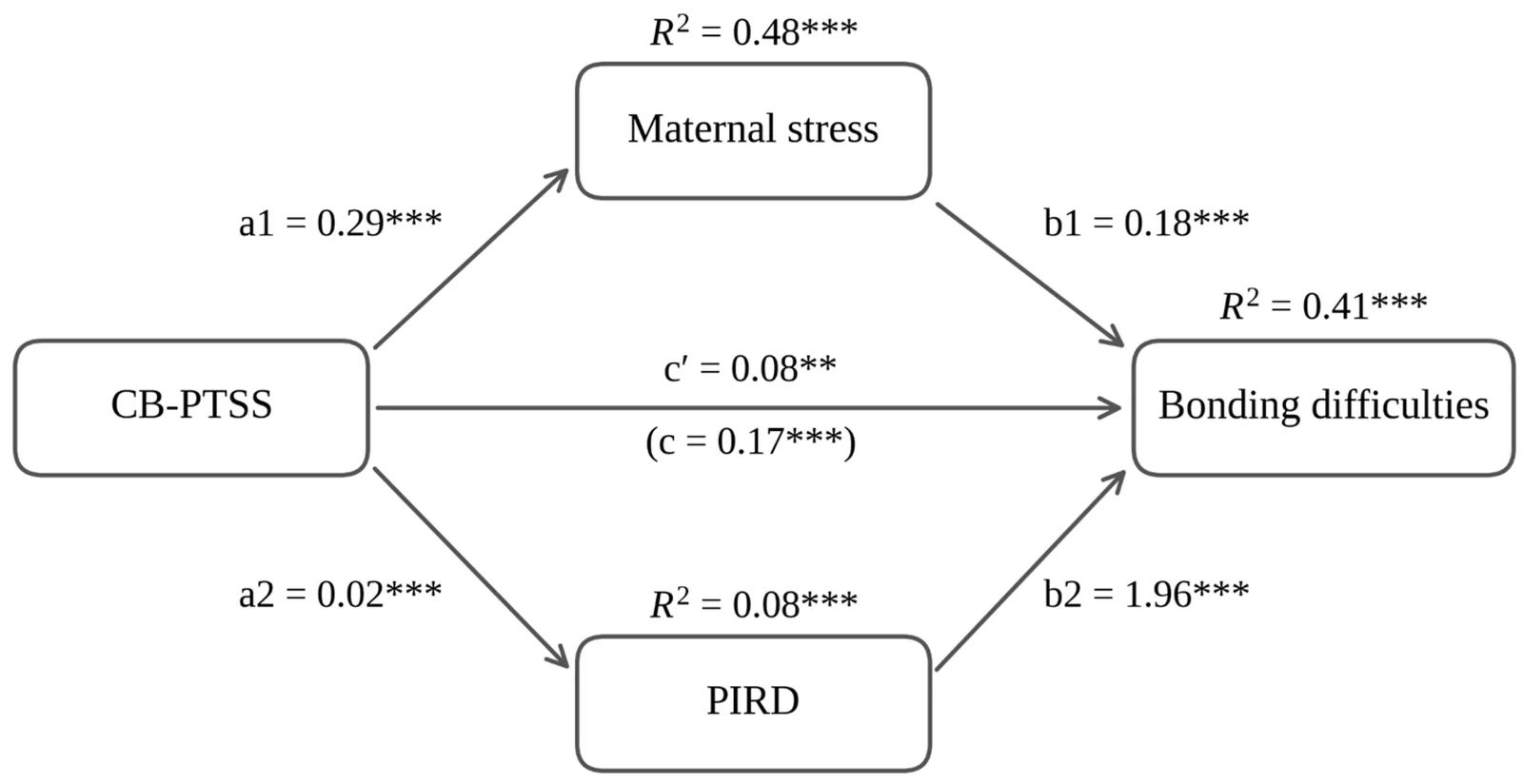
Parallel Mediation Model of the Association Between Childbirth-Related Posttraumatic Stress Symptoms and Mother–Infant Bonding Difficulties, via Maternal Stress and Perceived Infant Regulatory Difficulties (*N* = 534). Note. CB-PTSS = childbirth-related posttraumatic stress symptoms; PIRD = perceived infant regulatory difficulties. Values are unstandardized path coefficients (B), adjusted for maternal age, educational level, household income, infant age, and previous psychological problems. c′ = direct effect of CB-PTSS on bonding difficulties; c = total effect (in parentheses). R^2^ values indicate the variance explained in each outcome variable. ** *p* < 0.01, *** *p* < 0.001.

**Table 1:** Sociodemographic and Obstetric Characteristics of Participants (*N* = 542).

Variable	*n* (%) or *M* (*SD*)
Maternal age (years)	32.76 (5.03)
Education	
Elementary/High school	202 (37.3)
University/Postgraduate degree	340 (62.7)
Marital status	
Married/in a relationship	511 (94.3)
Single/separated/divorced	31 (5.7)
Household monthly income	
<€1583	276 (50.9)
≥€1583	266 (49.1)
History of psychological problems	1.95 (0.70)
Parity	
Primiparous	344 (63.8)
Multiparous	195 (36.2)
Difficulty conceiving (yes)	130 (24.0)
Infant age (months)	8.56 (6.47)
Gestational age (weeks)	38.89 (1.54)
Type of delivery	
Vaginal delivery	283 (52.2)
Elective/scheduled cesarean	95 (17.5)
Emergency cesarean	100 (18.5)
Instrumented vaginal delivery	112 (20.7)
Obstetric complications (yes)	307 (56.6)

*Note*. Percentages are based on valid responses; *N* varies slightly across variables due to missing data (*n* = 539 for parity, *n* = 534 for infant age). History of psychological problems was assessed on a 5-point scale (1 = totally disagree to 5 = totally agree), with greater scores indicating greater endorsement of prior psychological problems.

**Table 2:** Descriptive Statistics and Bivariate Correlations Among Study Variables and Covariates (*N* = 542).

Variable	*M*	*SD*	1	2	3	4	5	6	7	8
1. CB-PTSS	8.84	7.88								
2. Maternal stress	4.86	4.04	0.66 ***							
3. PIRD	2.15	0.92	0.24 ***	0.24 ***						
4. Bonding difficulties	4.43	4.05	0.38 ***	0.40 ***	0.54 ***					
5. Maternal age			−0.06	0.01	−0.01	0.07				
6. Educational level ^a^			0.01	0.06	−0.01	0.16 ***	0.34 ***			
7. Household income ^b^			−0.05	−0.01	0.01	0.17 ***	0.21 ***	0.38 ***		
8. Infant age			0.04	0.09 *	0.09 *	0.11 **	0.10 *	0.04	0.05	
9. Previous psychological problems			0.43 ***	0.44 ***	0.22 ***	0.24 ***	−0.08	−0.12 **	−0.14 **	−0.01

*Note*. CB-PTSS = childbirth-related posttraumatic stress symptoms; PIRD = perceived infant regulatory difficulties. Descriptive statistics for covariates are reported in Table 1. ^a^ Coded 0 = no university degree, 1 = university degree. ^b^ Coded 0 = below €1583, 1 = €1583 or higher. *n* = 534 for correlations involving infant age. * *p* < 0.05. ** *p* < 0.01. *** *p* < 0.001.

**Table 3:** Hierarchical Regression Analysis Predicting Mother–Infant Bonding Difficulties (*N* = 534).

Predictor	*B*	*SE B*	*β*	*t*	*p*	*R* ^2^	*R* ^2^ * Adj.*
Block 1							
Maternal age	0.01	0.04	0.01	0.25	0.803	0.12	0.11
Educational level	1.07	0.38	0.13	2.79	0.005		
Household income	1.18	0.36	0.15	3.27	0.001		
Infant age	0.06	0.03	0.10	2.49	0.013		
Previous psychological problems	1.59	0.24	0.27	6.63	<0.001		
Block 2							
Maternal age	0.02	0.03	0.02	0.53	0.595	0.23	0.22
Educational level	0.74	0.36	0.09	2.06	0.040		
Household income	1.22	0.34	0.15	3.62	<0.001		
Infant age	0.05	0.02	0.07	1.92	0.055		
Previous psychological problems	0.50	0.26	0.09	1.93	0.054		
CB-PTSS	0.10	0.03	0.20	3.84	<0.001		
Maternal stress	0.22	0.05	0.22	4.20	<0.001		
Block 3							
Maternal age	0.02	0.03	0.02	0.58	0.565	0.41	0.40
Educational level	0.84	0.32	0.10	2.64	0.009		
Household income	1.06	0.30	0.13	3.59	<0.001		
Infant age	0.03	0.02	0.04	1.20	0.229		
Previous psychological problems	0.18	0.23	0.03	0.79	0.427		
CB-PTSS	0.08	0.02	0.15	3.26	0.001		
Maternal stress	0.18	0.05	0.18	3.77	<0.001		
PIRD	1.95	0.15	0.44	12.60	<0.001		

*Note*. CB-PTSS = childbirth-related posttraumatic stress symptoms; PIRD = perceived infant regulatory difficulties. Block 1: F(5, 528) = 14.57, *p* < 0.001. Block 2: F(7, 526) = 22.78, *p* < 0.001. Block 3: F(8, 525) = 45.77, *p* < 0.001. All tolerance values > 0.10 and VIF values < 5.

**Table 4:** Full Model Coefficients for the Parallel Mediation of CB-PTSS on Mother–Infant Bonding Difficulties via Maternal Stress and Perceived Infant Regulatory Difficulties, Adjusted for Covariates (*N* = 534).

Predictor	*B*	*SE*	*t*	*p*	95% CI [LL, UL]
*Outcome: Maternal stress, R* ^2^ * = 0.48, F(6, 527) = 79.84, p < 0.001*					
CB-PTSS (a1)	0.29	0.02	160.24	<0.001	[0.257, 0.328]
Education	0.16	0.15	10.11	0.267	[−0.124, 0.446]
Household income	0.05	0.08	00.58	0.563	[−0.116, 0.214]
Maternal age	0.03	0.03	00.94	0.347	[−0.027, 0.077]
Infant age	0.04	0.02	20.07	0.039	[0.002, 0.080]
Previous psychological problems	10.22	0.21	50.94	<0.001	[0.814, 10.618]
*Outcome: Perceived infant regulatory difficulties, R* ^2^ * = 0.08, F(6, 527) = 7.61, p < 0.001*					
CB-PTSS (a2)	0.02	0.01	30.67	<0.001	[0.009, 0.031]
Education	0.02	0.04	00.53	0.600	[−0.063, 0.109]
Household income	−0.01	0.03	−00.31	0.733	[−0.059, 0.041]
Maternal age	0.00	0.01	00.07	0.950	[−0.015, 0.016]
Infant age	0.01	0.01	20.02	0.044	[0.000, 0.024]
Previous psychological problems	0.19	0.06	30.07	0.002	[0.068, 0.311]
*Outcome: Bonding difficulties, R* ^2^ * = 0.41, F(8, 525) = 45.52, p < 0.001*					
CB-PTSS (c′, direct effect)	0.08	0.02	30.29	0.001	[0.032, 0.124]
Maternal stress (b1)	0.18	0.05	30.76	<0.001	[0.084, 0.267]
PIRD (b2)	10.96	0.15	120.68	<0.001	[10.655, 20.262]
Education	0.57	0.16	30.69	<0.001	[0.268, 0.877]
Household income	0.20	0.09	20.18	0.030	[0.020, 0.372]
Maternal age	0.02	0.03	00.63	0.532	[−0.038, 0.073]
Infant age	0.03	0.02	10.34	0.183	[−0.013, 0.070]
Previous psychological problems	0.18	0.23	00.79	0.433	[−0.268, 0.625]
*Bootstrapped indirect effects (5000 bias-corrected bootstrap resamples)*					
**Indirect Effect**	***B***	***BootSE***			**95% BootCI [LL, UL]**
Total indirect effect	0.09	0.02			[0.050, 0.131]
Via maternal stress	0.05	0.02			[0.020, 0.084]
Via PIRD	0.04	0.01			[0.016, 0.064]

*Note*. CB-PTSS = childbirth-related posttraumatic stress symptoms; PIRD = perceived infant regulatory difficulties. Total effect of CB-PTSS on bonding difficulties (c) = 0.17, SE = 0.02, t = 7.54, *p* < 0.001, 95% CI [0.125, 0.212]. Indirect effects are considered statistically significant when the 95% bootstrap confidence interval does not include zero.

## Data Availability

The datasets generated and/or analysed during the current study are available from the corresponding author on reasonable request.
